# Aging without inflammaging: lesson from *Spalax*

**DOI:** 10.18632/aging.103953

**Published:** 2020-08-27

**Authors:** Irena Manov, Amani Odeh, Imad Shams

**Affiliations:** 1 University of Haifa, Haifa, Israel

**Keywords:** *Spalax*, cellular senescence, senescence‐ associated secretory phenotype (SASP), aging, inflammaging

Senescent cells are unable to divide and, thus, they possess a barrier for transmission of mutations for the next generations of cells. However, senescent cells remain alive and reportedly accumulate in tissues with age and, noteworthy, exhibit increased secretion of inflammatory cytokines, chemokines and growth factors (senescent secretome or senescent-associated secretory phenotype, SASP). SASP appears to be beneficial early in life by contributing to embryonic development, wound healing and suppression of malignancy, but deleterious for aging organisms promoting sterile inflammation and leading to age-related disorders, including cancer. This is consistent with the theory of antagonistic pleiotropy of aging, which implies the contribution of pleiotropic genes that have evolved to maintain fitness in youth when selection is strong, but they become harmful when selection weakens with age and reproductive period is over (Reviewed in [1]).

However, a huge variety of species, living in various ecosystems and forced to adapt to environmental stress in order to survive and maintain reproduction, contributed to the selection of traits that are more likely to be an exception than the rule in the wild. Among mammals, the underground blind mole rat of Mediterranean region *Spalax* and African underground rodents (e.g. Damaraland mole rat, *Fukomys damarensis* and Naked mole rat, *Heterocephalus glaber*) spend their lives in deep underground nests, in an environment with low oxygen and high carbon dioxide contents [2,3]. For above-ground rodents, these conditions provoke toxic stress and may cause cellular damage. The ecological adaptation of such mammals in extreme environmental conditions has shaped stress-resistant mechanisms allowing them to survive under continuing stresses. Along with adapting to environmental stress, many of these mammals have also acquired cancer resistance and longevity. The evolutionary forces primarily developed effective DNA repair to maintain genomes of cancer-resistant and long-lived mammals as one of the main factors of resistance to stress.

We focus on studying the molecular mechanisms of hypoxia tolerance, cancer resistance, and aging in *Spalax*, the Mediterranean blind mole rat. *Spalax* is one of the “champions” of longevity, which lives in captivity for ~ 17-20 years and does not exhibit an obvious aging phenotype, while maintaining physical and digging activity without signs of sarcopenia, frailty, hair loss and other signs of aging. Our recent report demonstrated a unique mechanism of cellular senescence in *Spalax* fibroblasts, which, like senescent human and mouse fibroblasts, showed proliferative arrest, SA-β-Gal-positive staining and increased expression of p21 and p53; however, signs of cellular senescence in *Spalax* were not accompanied by the secretion of the canonical inflammatory factors (Interleukins IL6, IL8, IL1α, SerpinB2, GROα, ICAM-1) both after replicative senescence and etoposide-induced senescence [4]. Noteworthy is the finding that the observed suppression of pro-inflammatory signals was not limited only to *Spalax* cultured cells: down-regulation of mRNA expression of pro‐inflammatory SASP representatives was also found in aging *Spalax* tissues. These results strongly support the notion that suppressed inflammatory response is a general *Spalax* evolutionary strategy that does not follow the theory of antagonistic pleiotropy of aging.

Cellular senescence and SASP are controlled directly by persistent DNA damage pathway (Reviewed in [5]). Since DNA damage in *Spalax* during senescence is transient (due to effective DNA repair [4,6]) and does not reach a persistent level, the inflammatory response is suppressed. However, effective DNA repair is not the only mechanism that determines the downregulation of SASP in *Spalax*. The positive regulation of feedback loop of IL1α-NF‐κB appears to be impaired in *Spalax*. Previous research has shown that the IL1 pathway acts as an upstream regulator of SASP [7], and inhibition of the IL1 receptor in senescent human embryonic lung fibroblasts IMR90 resulted in inhibition of SASP without affecting the cell cycle arrest associated with senescence [8]. In our experiments, suppression of IL1 receptor by a chemical antagonist decreased IL6 secretion in *Spalax* cells, which was activated by an exogenous inflammatory stimulus [4].

Thus, the uncoupling of inflammatory phenotype and cellular senescence provides an opportunity for independent study of these phenomena and creates a unique approach to the development of new inflammation inhibitors that can potentially be used to suppress inflammaging in the elderly and to inhibit of the cytokine storm in patients with the emerging COVID-19. In this context, the elucidation of the molecular mechanisms restraining the inflammatory response in *Spalax* seems to be very important. These strategies were developed and fine-tuned by evolutionary forces during millions of years facilitating longevity, healthy aging and reduced cancer risk (Figure 1).

**Figure 1:**
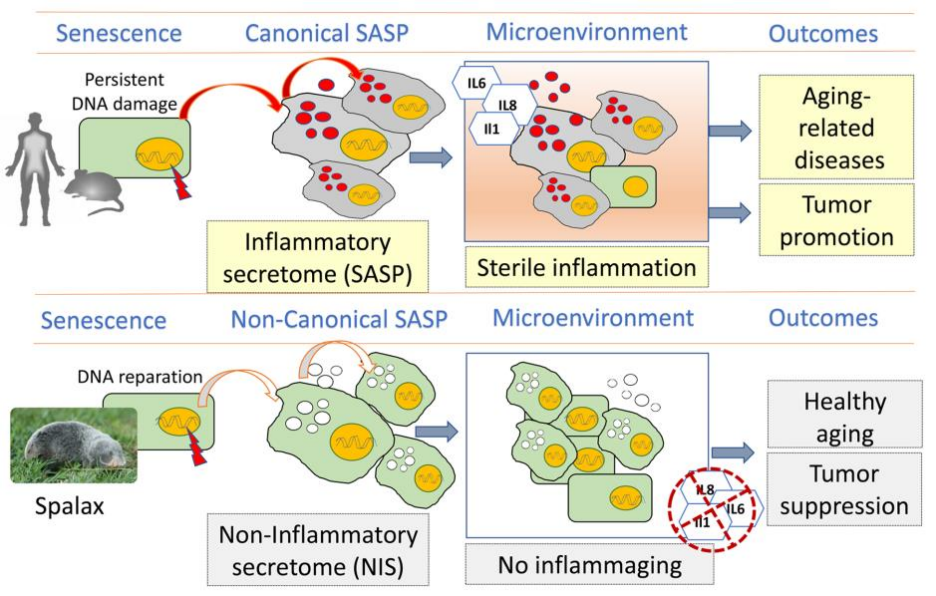
**Schematic diagram showing the differences between canonical inflammatory SASP (human / mouse) and non-inflammatory secretome (NIS) in *Spalax* and their effects on homeostasis in aging tissues.** Canonical secretome of senescent cells comprises a specific pattern of inflammatory mediators and growth factors that may induce senescence in surrounding cells, an effect known as "bystander senescence". The accumulation of senescent cells in aging leads to the amplification of SASP, which in turn modulates the surrounding tissues and causes the so-called "sterile inflammation"- a microenvironment that supports most age-related pathologies, including malignant neoplasms. Due to the efficient DNA repair and other mechanisms, *Spalax* senescent cells do not produce the main inflammatory factors that are involved in the development of age-related pathologies. Hence, when these cells transmit senescence to surrounding cells via the paracrine factors of "non-inflammatory secretome" (NIS), the recipient cells also do not elicit an inflammatory response and therefore cannot maintain "sterile inflammation" and cancer development in older *Spalax*.

## References

[1] Flatt T, Schmidt PS. Biochim Biophys Acta. 2009; 1790:951–62. 10.1016/j.bbagen.2009.07.010 19619612 PMC2972575

[2] Fang X, et al. Cell Rep. 2014; 8:1354–64. 10.1016/j.celrep.2014.07.030 25176646 PMC4350764

[3] Shams I, et al. Comp Biochem Physiol A Mol Integr Physiol. 2005; 142:376–82. 10.1016/j.cbpa.2005.09.003 16223592

[4] Odeh A, et al. Aging Cell. 2020; 19:e13045. 10.1111/acel.13045 31605433 PMC6974727

[5] da Silva PF, Schumacher B. Open Biol. 2019; 9:190168. 10.1098/rsob.190168 31744423 PMC6893400

[6] Domankevich V, et al. BMC Evol Biol. 2016; 16:177. 10.1186/s12862-016-0743-8 27590526 PMC5010716

[7] Orjalo AV, et al. Proc Natl Acad Sci USA. 2009; 106:17031–36. 10.1073/pnas.0905299106 19805069 PMC2761322

[8] Lau L, et al. Mol Cell Biol. 2019; 39:e00586-18. 10.1128/MCB.00586-18 30988157 PMC6549465

